# 

**Published:** 1898-12-17

**Authors:** 


**Notices of Births, Deaths, and Marriages** are inserted in this column at a charge of 2s. 6d. for an announcement not exceeding four lines.

### NOTICE TO CORRESPONDENTS.

All MS., Letters, Books for Review, and other matters intended for the Editor should be addressed The Editor, "The Hospital," The Hospital Building, 28 & 29, Southampton Street, Strand, W.C.

The Editor cannot undertake to return rejected MS., even when accompanied by stamped directed envelope.

**Notice to Advertisers and Subscribers.**

All Advertisements, Orders for Copies of The Hospital, and Business Communications should be addressed to **THE MANAGER (Not to the Editor)**, The Hospital, The Hospital Building, 28 & 29, Southampton Street, Strand, London, W.C.

The Cost of Subscription, post free, is as follows:—Per week, 2½d.; per quarter, 2s. 8d.; per half-year, 5s. 3d.; per annum, 10s. 6d. Foreign:—Per annum, 15s. 2d., payable, in all cases, in advance.

**NOTICE.—Vol. XXIV. of "The Hospital" (April, 1898, to September, 1898), in handsome cloth binding, is now on sale at the Office of this Journal, price 7s. 6d. Cases for binding the Numbers contained in this Volume 1s. 6d. Index to Vol. XXIV., 2½d., post free.**

**The Monthly Part for December is now ready. Price 6d., by post 8d.**

### BOOKS RECEIVED.

Kegan Paul, Trench, and Co.

"General Nursing." By Eva C. E. Lückes.

Eason and Son, Dublin.
"Every Hour Diaries, 1899."

Cassell and Co.

"Letts' Medical Diary, 1899."

Adam and Charles Black.
"Who's Who, 1899." By Douglas Sladen.

**Periodicals and Pamphlets.**—Literary Digest, Boy's Own Paper (Christmas Number), Girl's Own Paper (Supplement), Sunday at Home, Leisure Hour, Leeds Hospital Magazine, London, Medical Record, Woman's Signal, American Archæologist, Science.

### SWEET CHARITY'S GUIDE TO CHRISTMAS GIVERS.

Pressure upon the space in our Christmas Supplement compels us to insert the following additional list of appeals below:—

**West-end Hospital for Paralysis, Epilepsy, &c**, 73, Welbeck Street, W.—A special appeal is now being made to enable the committee of this institution to pay off a debt of £6,000 incurred in rebuilding. Treasurer, Mr. H. Alexander Dowell.

**Cancer Hospital**, Fulham Road, S.W.—This institution is free to poor persons suffering from cancerous disease. There are a number of beds provided for the use of patients who may remain for life. Patients are received not from London only, but from all parts of Great Britain as well as from the Colonies. Assistant Secretary, Mr. C. Jarman.

**Central London Ophthalmic Hospital, Gray's Inn Road.**—Owing to the inadequacy of the building to meet the increased demands for aid, efforts are being made to obtain funds for the enlargement and gradual reconstruction of the hospital, towards which over £2,000 has already been subscribed. Secretary, Mr. J. G. Bryant.

**Royal Society for the Prevention of Cruelty to Animals**, 105, Jermyn Street, S.W.—The work of this society is too well known to need lengthy comment. The increased operations of late years have drawn from the funds an amount largely exceeding the yearly subscriptions. Assistance may be rendered in two ways—first, by supplying the society with information concerning acts of cruelty, and secondly by increasing the revenue by means of annual subscriptions or donations. Secretary, Mr. John Colam.

**Sea-side Convalescent Hospital, Seaford, Sussex.**—At this, the first established charity of its class in England, 1,153 patients received its benefits last year. The institution is unendowed, and there is no reserve fund of any kind. Funds are seriously crippled, and £1,000 is wanted before Christmas to meet the liabilities. Secretary's office, 430, Strand, W.C.

### The Student of Medicine.

**APPOINTMENTS.**

F. W. Bailey, M.R.C.S.Eng., L.R.C.P.Lond., appointed Honorary Anæsthetist to the Liverpool Dental Hospital. J. B. Bate, L.S.A., appointed Resident Medical Officer at the Mogden Isolation Hospital. R. W. Bollans, M.B., Ch.B.Vict., appointed Medical Officer for the Hadfield District of the Glossop Union. E. C. Bousfield, L.R.C.P.Lond., M.R.C.S.Eng., appointed Bacteriologist for the Parish of Camberwell. E. J. Bray, M.B.Edin., appointed Medical Attendant on the Aborigines at Wingham, Manning River. T. H. Brown, M.B.Camb., appointed Junior House Surgeon to the Bradford Royal Infirmary. H. S. Collier, F.R.C.S., appointed Assistant Surgeon to the Hospital for Sick Children, Great Ormond Street. S. Connor, M.D., appointed Health Officer for Wannon Shire, Nareen Riding, Victoria. W. N. Davies, M.D.R.U.I., M.Ch., appointed Medical Officer for the Llantrissant and Llantwit Fardre District of the Pontypridd Union. J. F. Dobson, M.B.Lond., appointed Resident Casualty Officer to the General Infirmary, Leeds. A. Earnshaw, M.B.Lond., M.R.C.S., appointed House Physician to the Bradford Royal Infirmary. L. Evans, M.A., M.B., B.C.Cantab., appointed House Surgeon to the Royal Orthopædic Hospital, Oxford Street. F. R. Fairbank, M.D.Heidelb., F.R.C.S.Edin., M.R.C.S.Eng., appointed Medical Officer and Public Vaccinator for the Western District of the Dorking Union. J. M. Ferguson, L.R.C.P.E., L.R.C.S.Edin., appointed Medical Officer to the Burnley Joint Infectious Diseases Hospital. G. T. Hankins, M.R.C.S.Eng., L.S.A., appointed Surgeon for Diseases of the Nose, Ear, and Throat, Prince Alfred Hospital, Sydney. H. N. Harness, M.R.C.S.Eng., L.R.C.P.Lond., appointed Third Assistant Medical Officer to the Kent County Asylum, Chartham, near Canterbury, Kent. W. W. Hearne, M.B., appointed Resident Surgeon, Prince Alfred Hospital, Melbourne. J. P. Hocken, M.D., appointed Government Medical Officer and Vaccinator for the District of West Walsend, New South Wales. R. N. Lankister, M.B., B.Sc.Durh., appointed Dispensary Surgeon to the Bradford Royal Infirmary. W. T. Lister, B.A., M.B., B.C., F.R.C.S., appointed Assistant Surgeon to the Central London Ophthalmic Hospital. J. S. Low, M.B., B.Ch., B.Sc Edin., appointed, by the India Office, a Medical Officer for Special Plague Duty in India. E. McAnally, M.R.C.S., L.R.C.P., appointed Medical Officer for the Third District of the Hollingbourn Union. R. A. A. Manly, M.B., appointed Health Officer for the Shire of Wyndham, Victoria. L. A. Nolan, L.K.Q.C.P.I., &c., appointed Health Officer for the Shire of Rosedale, Rosedale and Denison Ridings, Victoria. Dr. Rawlings, appointed Medical Officer and Public Vaccinator for the Northern District of the Dorking Union. E. A. Roberts, M.D., appointed Medical Officer for the Twentieth District of the Kensington Union. D. Rice, L.R.C.P.Lond., M.R.C.S.Eng., appointed House Physician at Bethlem Royal Hospital. V. J. R. Robin, L.R.C.P.Lond., M.R.C.S.Eng., appointed Health and Medical Officer at Port Douglas, Queensland. R. U. Russell, L.R.C.S.I., appointed Port Health Officer for Newcastle, New South Wales. L. A. Smith, M.B.Lond., M.R.C.S.Eng., L.R.C.P.Lond., appointed Medical Registrar to the London Hospital, Whitechapel, E. A. E. Stevens, M.B.Durh., L.R.C.P.Lond., M.R.C.S., appointed House Physician to Bethlem Royal Hospital. W. H. B. Stoddart, M.B., M.R.C.P., appointed Assistant Medical Officer to the Bethlem Royal Hospital. E. C. Taylor, M.D., F.R.C.S., appointed Medical Officer to the Hampstead Workhouse. F. L. Underwood, M.R.C.S., L.R.C.P.Lond., appointed Medical Officer for the Third District of the Daventry Union. H. Vines, L.R.C.P.-Edin., M.R.C.S.Eng., appointed Resident Medical Officer and Public Vaccinator at Broome, and Quarantine Officer for the Port of Broome, West Australia.

### WORKS FOR NURSES.

**A MANUAL FOR HOSPITAL NURSES** AND OTHERS ENGAGED IN ATTENDING ON THE SICK. By EDWARD J. DOMVILLE, L.R.C.P., M.R.C.S. Eighth Edition. Crown 8vo., 2s. 6d.

**A MANUAL OF NURSING, MEDICAL AND SURGICAL.** By C. J. CULLINGWORTH, M.D., F.R.C.P. Third Edition. Fcap. 8vo., 2s. 6d.

**A SHORT MANUAL FOR MONTHLY NURSES.** By C. J. CULLINGWORTH, M.D. Fourth Edition. Revised by M. A. Atkinson. Fcap. 8vo., 1s. 6d.

**NOTES ON GYNÆCOLOGICAL NURSING.** By J. B. HELLIER, M.D. Fcap. 8vo., 1s. 6d.

**HINTS ON ELEMENTARY PHYSIOLOGY.** By FLORENCE A. HAIG-BROWN. With a Preface by Dr. Ord With 21 Illustrations. Fcap. 8vo., 1s. 6d.

**LECTURES ON MEDICINE TO NURSES.** By HERBERT E. CUFF, M.D. Second Edition. With 29 Illustrations. Crown 8vo., 3s. 6d.

**ANTISEPTIC PRINCIPLES FOR NURSES.** By C. E. RICHMOND, F.R.C.S. Fcap. 8vo., 1s.

**A SHORT DICTIONARY OF MEDICAL TERMS.** 2s. 6d.

**CHAVASSE'S ADVICE TO A MOTHER** ON THE MANAGEMENT OF HER CHILDREN, and on the Treatment on the Moment of their more Pressing Illnesses and Accidents. Fifteenth Edition by Dr. GEORGE CARPENTER, Physician to the Evelina Hospital for Sick Children. 2s. 6d.

**CHAVASSE'S ADVICE TO A WIFE ON** THE MANAGEMENT OF HER OWN HEALTH, and on the Treatment of Some of the Complaints incidental to Pregnancy, Labour, and Suckling. With an Introductory Chapter especially addressed to a Young Wife. Fourteenth Edition, revised by Dr. FANCOURT BARNES, Consulting Physician to the British Lying-in Hospital. 2s. 6d.

London: J. and A. CHURCHILL,
7, Great Marlborough Street.

